# Moral injury and mental health in healthcare workers are linked to organizational culture and modifiable workplace conditions: Results of a national, mixed-methods study conducted at Veterans Affairs (VA) medical centers during the COVID-19 pandemic

**DOI:** 10.1371/journal.pmen.0000085

**Published:** 2024-12-23

**Authors:** Natalie Purcell, Daniel Bertenthal, Hajra Usman, Brandon J. Griffin, Shira Maguen, Sarah McGrath, Joanne Spetz, Sylvia J. Hysong, Haley Mehlman, Karen H. Seal

**Affiliations:** 1 San Francisco Veterans Affairs Health Care System, San Francisco, San Francisco, California, United States of America; 2 Department of Social and Behavioral Sciences, University of California, San Francisco, San Francisco, California, United States of America; 3 Northern California Institute for Research and Education, San Francisco, San Francisco, California, United States of America; 4 Central Arkansas VA Health Care System, Center for Mental Health Care and Outcomes Research, North Little Rock, Arkansas, United States of America; 5 University of Arkansas for Medical Sciences, Psychiatric Research Institute, Little Rock, Arkansas, United States of America; 6 Department of Psychiatry and Behavioral Sciences, University of California, San Francisco, San Francisco, California, United States of America; 7 Philip R. Lee Institute for Health Policy Studies, School of Medicine, University of California, San Francisco, San Francisco, California, United States of America; 8 Center for Innovations in Quality, Effectiveness and Safety, Michael E. DeBakey Veterans Affairs Medical Center, Houston, Texas, United States of America; 9 Department of Medicine—Health Services Research Section, Baylor College of Medicine, Houston, Texas, United States of America; 10 Department of Psychology, University of Michigan, Ann Arbor, Michigan, United States of America; 11 Department of Medicine, University of California, San Francisco, San Francisco, California, United States of America; Kanagawa University of Human Services, JAPAN

## Abstract

Using mixed methods, we examined drivers of risk for moral injury, mental health symptoms, and burnout among frontline healthcare workers in high-risk Veterans Affairs (VA) clinical settings during the COVID-19 pandemic. Across 21 VA medical centers, 2,004 healthcare workers completed an online survey assessing potential risk factors for moral injury, posttraumatic stress, depression, and burnout. Assessed risk factors included: pandemic exposures; individual worker characteristics; aspects of workplace/organizational culture; and facility performance on standardized measures of care quality, patient satisfaction, and employee satisfaction (extracted from VA administrative data). Among surveyed workers, 39% were at risk for moral injury, 41% for posttraumatic stress, 27% for depression, and 25% for persistent burnout. In generalized linear mixed models, significant predictors of moral injury risk included perceived lack of management support for worker health/safety, supervisor support, coworker support, and empowerment to make job-related decisions—all modifiable workplace factors. Pandemic-related risk factors for moral injury included prolonged short-staffing, denying patient-family visits, and high work-family conflict. Predictors of posttraumatic stress, depression, and burnout were similar. Forty-six surveyed workers completed a follow-up qualitative interview about experiences of moral distress in the workplace, and interview themes aligned closely with survey findings. Rapid qualitative analysis identified protective factors that may reduce moral injury risk, including a collaborative workplace community, engaged leadership, empowerment to make changes in the workplace, and opportunity to process distressing events. We conclude with recommendations to mitigate moral injury risk in healthcare organizations. These include involving workers in discussions of high-stakes decisions that will affect them, creating consistent and clear channels of communication between the frontlines and leaders of the organization, practicing leadership rounding to improve leaders’ understanding of the daily work of frontline teams, and collaborating to understand how existing processes and policies may contribute to safety risks and moral conflict.

## Introduction

Moral injury is an enduring psychological and spiritual distress that can result from violating one’s values [1,2] or experiencing the betrayal of those values by a trusted authority [3]. In decades past, the term was used almost exclusively to describe the experiences of combat veterans. But as scientific literature about moral injury grew so too did recognition that civilians could be at risk, especially workers in high-stakes, high-stress roles such as healthcare providers and first responders [4].

Healthcare workers can face a variety of morally challenging and potentially distressing situations at work—for example, time constraints that affect care relationships and quality, social inequities that create care disparities, lack of personnel or resources needed for optimal care, difficult decisions to triage care, and professional responsibility in situations where errors could have serious consequences [4]. During the COVID-19 pandemic, these factors were exacerbated and new distressing experiences became commonplace. These included high rates of hospitalization and death, shortages of supplies and equipment that could jeopardize care quality, policies requiring isolation of critically ill patients from loved ones, and working conditions that put healthcare workers and their families at elevated risk of COVID-19 [5–8].

Significant or cumulative experiences of moral distress in the healthcare workplace—that is, unsettling conflicts between one’s values and one’s actions or experiences—can lead to the lasting psychological and spiritual wound of moral injury [9,10]. Moral injury is characterized by enduring feelings of guilt, shame, and disillusionment; these are often linked with low self-esteem, alienation, and isolation from others. Those experiencing moral injury may feel numb, demoralized, and lose ambition; they may feel they do not deserve to be happy or have a good life. Moral injury is associated with posttraumatic stress disorder (PTSD), depression, anxiety, substance use, self-harm, and suicide [1,4,11,12].

The COVID-19 pandemic spurred a proliferation of studies on moral injury among healthcare workers [9,13]. Recent publications have examined the prevalence of moral injury, the workplace events that may trigger moral injury, and how moral injury negatively impacts workers’ mental health. Among frontline healthcare workers, moral injury prevalence estimates during the pandemic have ranged from approximately 27% to 46% [8,13–16]. Pandemic-era studies have found similarly high rates of other mental health problems among healthcare workers, including PTSD, depression, and anxiety, which are often correlated with moral injury [13,14]. Researchers have identified pandemic-related exposures such as caring for patients dying of COVID-19 and lack of personal protective equipment as contributors to moral injury and poor mental health outcomes.

Yet gaps remain in our understanding of moral injury among healthcare workers: Which healthcare workers and settings are at greatest risk for moral injury and why? What experiences and events lead to moral injury among healthcare workers? And, finally, what workplace conditions and organizational factors make moral injury more or less likely? Addressing these questions is necessary to inform policies, procedures, and environmental changes that reduce the risk of moral injury in healthcare.

In 2023, Griffin and colleagues [4] proposed a “contextual dimensional” theory of moral injury that can guide a systematic approach to addressing these and related questions. Informed by a review of 185 articles focused on moral injury in healthcare workers, they theorized that moral injury is an individual syndrome with causal roots in societal, organizational, interpersonal, individual, and event-specific contextual factors. Taken together, these factors can interact to mitigate, exacerbate, or complicate moral injury risk. During the COVID-19 pandemic, for example, health care workers could be exposed to situations in which doing right by one set of ethical standards (e.g., organizational guidelines about efficient use of resources) required transgression of another (e.g., personal commitment to prioritizing each individual patient’s chance of survival). Following Griffin and colleagues, a holistic and multilevel analysis of moral injury among healthcare workers requires assessment of exposure to potentially morally injurious events as well as possible risk factors residing within the healthcare workplace (societal/organizational), the team (interpersonal), and the worker (individual).

In alignment with this contextual theory of moral injury, we conducted a mixed-methods study to examine moral injury risk among frontline VA healthcare workers in high-risk clinical settings during the COVID-19 pandemic. Integrating VA administrative data, survey methods, and in-depth qualitative interviews across a large, nationwide sample, we sought a more comprehensive understanding of how moral injury risk is related to pandemic exposures and events, individual worker characteristics, workplace/organizational culture, and facility-level performance on measures of care quality, patient safety, patient satisfaction, and staff satisfaction. Our secondary objective was to assess risk for posttraumatic stress, depression, and burnout, and to examine how these relate to moral injury risk.

## Methods

### Study design

**Fig 1:**
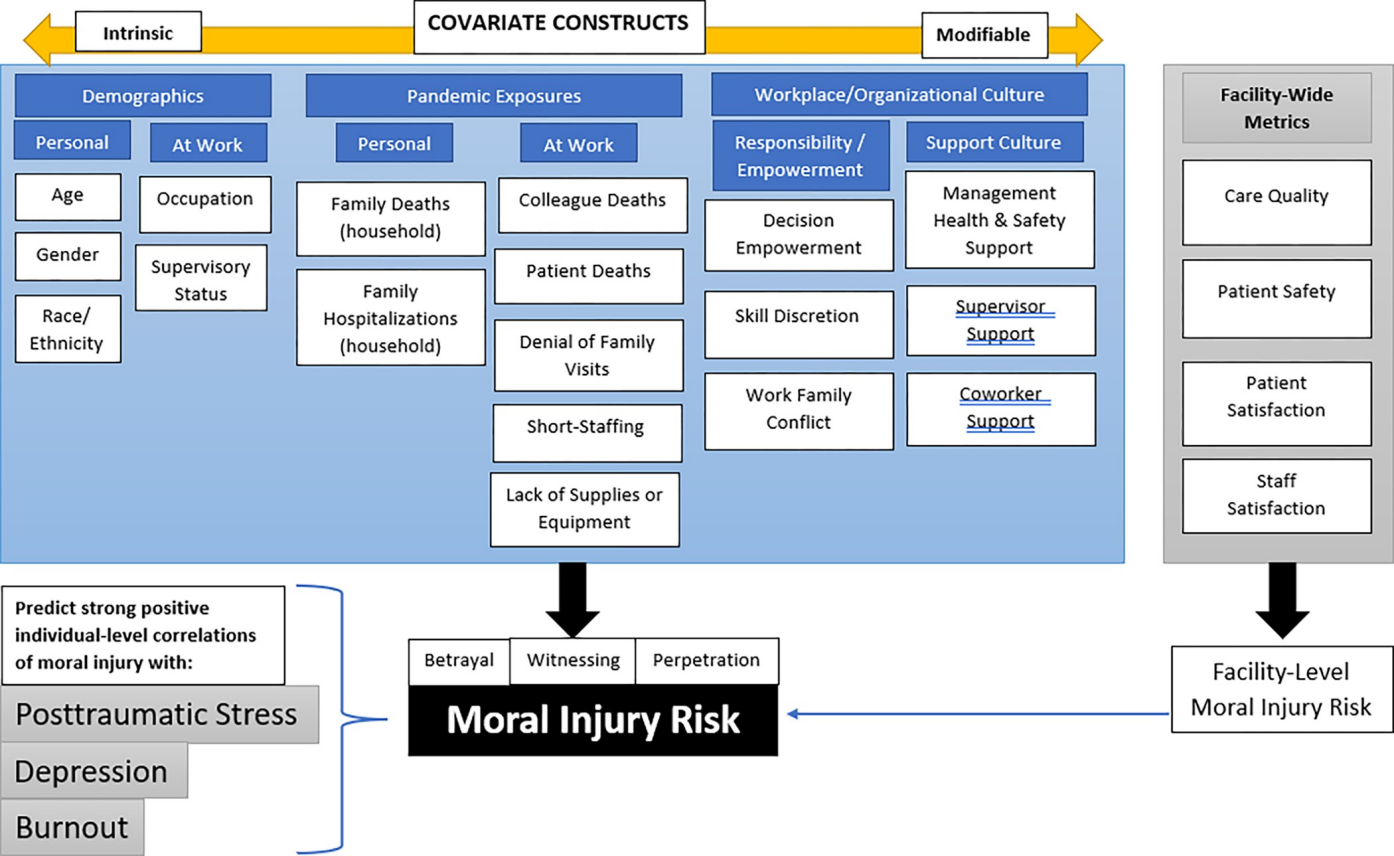
Conceptual model of factors affecting moral injury risk.

Informed by a contextual approach to moral injury [e.g., 4], we developed an initial conceptual model to guide our mixed-methods study design and our selection of data sources. Specifically, we hypothesized that moral injury risk factors include: (1) pandemic exposures and events, (2) individual worker characteristics, (3) workplace/organizational culture, and (4) facility-level care quality, safety, and satisfaction metrics. In each of these four domains, we specified variables with a potential relationship to risk for moral injury, including moral injury based (a) *betrayal* by the organization, colleagues, or others, (b) *witnessing* others’ unethical actions in the workplace, and (c) *perpetrating* unethical actions through acts of omission or commission [17,18]. We also hypothesized that moral injury risk (the primary outcome) would be correlated with risk for posttraumatic stress, depression, and burnout (secondary outcomes). The initial conceptual model is presented in Fig 1.

Multiple methods were necessary to gain a comprehensive understanding of how covariates of interest could affect risk of moral injury and secondary outcomes. Therefore, we designed the study to integrate VA administrative data, nationwide survey data, and qualitative interview data through all phases of the project.

### Setting

To ensure adequate pandemic-related event exposure, the recruitment pool consisted of all Veterans Affairs (VA) healthcare systems in the highest quartile of COVID-19 deaths per bed at the time the study was launched in 2021. The Directors and Research Chiefs for all VA healthcare systems in this pool were contacted by e-mail to invite their facilities to participate in the study. Of the 34 facilities contacted, 21 elected to participate in the project (62% facility participation rate). Participating healthcare systems spanned a wide geographic area, with multiple facilities from each major US region, including the Northeast, Southeast, Southwest, Midwest, Rocky Mountains, and Pacific Coast. Within each facility, inpatient units, emergency departments, and nursing homes were specifically targeted for inclusion. We chose these locations because we hypothesized that they would be at highest risk for moral injury and at highest risk for the pandemic-related exposures of interest.

### Quantitative methods

#### Quantitative instruments and measures

An online survey was developed in VA REDCap [19] to measure all outcomes and covariates of interest, excluding facility-level metrics. The survey was designed to be completed online in 15 minutes.

We assessed moral injury risk using the 9-item Moral Injury Events Scale (MIES) with minor modifications to ensure applicability to healthcare workers [20]. The MIES is not a direct measure of moral injury, but a proxy thereof; it assesses exposure to potentially morally injurious events and distress caused by that exposure. We defined a positive MIES screen as moderate, strong, or very strong agreement with two or more items (indicative of *both* exposure and, where applicable, *current* distress) in any one MIES domain—betrayal, witnessing, and/or perpetration. We considered those who screened positive on MIES to be *at risk for moral injury*. Other studies have dichotomized the MIES in a similar manner but have not required multiple items for a positive screen [cf. 17]. We chose to require multiple items within a single domain (including, where applicable, items indicating present distress) in order to use the MIES as a surrogate measure of risk for moral injury.

We assessed posttraumatic stress using the 6-item Primary Care PTSD Screener for DSM-5 [21]; positive screen was defined as confirmation of exposure to a traumatic event plus agreement with two or more subitems. We assessed depression using the 2-item Patient Health Questionnaire-2 [22]; positive screen was defined as a score of at least 3/6. We assessed burnout using a single-item burnout measure found to be highly correlated with the longer Maslach Burnout Inventory [23]; positive screen was defined as endorsing persistent symptoms that won’t go away on the single-item burnout measure. Across these measures, positive screens do not necessarily indicate clinically meaningful symptoms or the presence of any disorder. However, we can consider those who screened positive as *at risk* for the measured condition.

We assessed pandemic exposures using questions developed by the study team to measure level of exposure to patient deaths, coworker deaths, denial of family visits to critically ill patients, perceived short-staffing and duration thereof, and lack of supplies and equipment. Individual covariates were assessed using standard demographic questions (age, gender, race/ethnicity) and questions from the VA’s All Employee Survey (occupation, supervisory status) [24]. Workplace culture covariates (e.g., management health/safety support, supervisor support, coworker support, decision authority) were assessed using selected scales from the Centers for Disease Control and Prevention’s National Healthy Worksite Program Health & Safety Climate Survey: INPUTS [25,26].

We assessed facility-level covariates (care quality, patient safety, patient satisfaction, and staff satisfaction metrics) by extracting VA administrative data from multiple sources, including VA’s Strategic Analytics for Improvement and Learning; the VA All Employee Survey; and VA Human Resources data [27]. See Table 1 for a description of all instruments and measures.

**Table 1:** Instruments and measures for all outcomes and covariates.

Construct Label	Sample Item(s)	Instrument
**OUTCOMES**		
Moral Injury – Any	I am troubled by having acted in ways that violated my own morals or values.	MIES (9 items)
Moral Injury - Perpetration Domain	I am troubled because I violated my morals by failing to do something that I felt should have been done.	MIES (4 of 9 items)
Moral Injury - Witnessing Domain	I am troubled by having witnessed others’ immoral acts.	MIES (2 of 9 items)
Moral Injury - Betrayal Domain	I feel betrayed by leaders who I once trusted.	MIES (3 of 9 items)
PTSD	In the past month, have you felt guilty or unable to stop blaming yourself or others for the event(s) or any problems the event(s) may have caused?	Primary Care PTSD Screen for DSM-5
Depression	In the past month, how often have you been bothered by feeling down, depressed, or hopeless?	PHQ-2
Burnout	The symptoms of burnout that I’m experiencing won’t go away. I think about frustration at work a lot.	Single-Item Burnout Measure (West et al)
**COVARIATES**		
***Job & Unit Culture - Support Culture***		
Management Health & Safety Support	In this facility, management considers workplace health and safety to be important.	INPUTS
Supervisor Support	My supervisor is helpful in getting the job done.	INPUTS
Coworker Support	The people I work with can be relied on when I need help.	INPUTS
***Job & Unit Culture - Responsibility & Empowerment***		
Work-Family Conflict	How often do things going on at work make you feel tense and irritable at home?	INPUTS
Decision Empowerment	My job allows me to make a lot of decisions on my own.	INPUTS
Skill Discretion	My job requires me to be creative.	INPUTS
***Pandemic Exposures - At Work***		
Colleague Deaths	Someone you worked with died with from COVID-19.	Original
Patient Deaths	A patient you cared for died from COVID-19.	Original
Denial of Family Visits	You had to deny family visits for a critically ill veteran.	Original
Short-Staffing	Your team was short-staffed or lacked personnel needed for patient Care.	Original
Lack of Supplies or Equipment	You had difficulty obtaining needed supplies or equipment for yourself, your coworkers, or your patients.	Original
***Pandemic Exposures – Personal***		
Family Deaths	Someone you lived with died from COVID-19.	Original
Family Hospitalizations	Someone you lived with was hospitalized with COVID-19.	Original
***Demographics - At Work***		
Occupation	Which of the following best describes your occupation during the COVID-19 Pandemic?	VA AES
Supervisory Status	What is your level of supervisory responsibility?	VA AES
Work Location	Which of the following best describes your work location during the COVID-19 Pandemic?	VA AES
***Demographics – Personal***		
Age	What is your age?	Original
Gender	What is your gender?	Original
Race/Ethnicity	What is your race?	Original
**FACILITY-WIDE METRICS**		
Patient Safety / Care Quality	Acute Care 30-Day Standardized Mortality Ratio [SMR30]	VA SAIL
	All Cause Hospital-Wide 30-Day Readmission Rate	VA SAIL
	Patient Safety Index [PSI90]	VA SAIL
Patient Satisfaction	Patient-Centered Care Medical Homes [PCMH] Care Coordination	VA SAIL
	Hospital Consumer Assessment of Healthcare Providers and Systems [HCAHPS] Care Transition	VA SAIL
Employee Satisfaction	Personal Safety During the Pandemic Score	VA AES
	Best Places to Work Score	VA AES
	Organization Satisfaction Score	VA AES
	RN Turnover Rate	VA HR Data

#### Quantitative participants and procedures

Aided by local contacts at participating facilities and facility directories, we assembled a roster of e-mail addresses for all employees (regardless of occupation) working in inpatient units, emergency departments, and nursing homes at each facility. In assembling e-mail rosters, we prioritized inclusiveness over accuracy, which resulted in the inclusion of some healthcare workers whose primary work location was outside of the targeted high-risk departments.

We e-mailed survey invitations and up to three reminders to potential participants. To minimize volunteer bias, the e-mail invitation described the study as an evaluation of healthcare workers’ experiences during the COVID-19 pandemic without mentioning specific outcomes of interest. Survey administration dates ranged from 12/10/2021 to 6/1/2022, roughly corresponding with the Omicron wave of the COVID-19 pandemic (after widespread vaccine availability). Of 10,563 staff invited, 2,810 (26.6%) participated. Complete data were obtained for 2,004 participants.

Demographic characteristics and occupations of the 2,004 survey participants who were included in the analysis are presented in Table 2. Nurses constituted a majority of the sample with other healthcare occupations adequately represented. The sample was diverse with regard to race/ethnicity, gender, and age. Among participants included in the analysis, 80% (1,605) endorsed working in one of the high-risk work locations targeted for inclusion (i.e., intensive care unit, other inpatient unit, emergency department, or nursing home).

**Table 2:** Characteristics of 2,004 participants.

Characteristic	Summary
Age	
29 and under	105 (5.2%)
30-39	432 (21.6%)
40-49	597 (29.8%)
50-59	591 (29.5%)
60 years or older	279 (13.9%)
Occupation	
Registered nurse	1,103 (55.0%)
LPN/LVN or nursing asst.	341 (17.0%)
Nurse practitioner or physician asst.	65 (3.2%)
Physician	206 (10.3%)
Psychologist or social worker	42 (2.1%)
Other hands-on patient care (including respiratory therapists)	171 (8.5%)
Admin in clinical area, housekeeping, or other	76 (3.8%)
Supervisory status	
Non-supervisor	1,806 (90.1%)
Supervisor	198 (9.9%)
Gender	
Man	481 (24.0%)
Woman	1,464 (73.1%)
Non-binary	8 (0.4%)
Other or Declined	51 (2.5%)
Identifies as transgender	
Yes	12 (0.6%)
No or Declined	1,953 (99.4%)
Race and Ethnicity (*multi-select option*)	
African American or Black	319 (15.9%)
American Indian or Alaska Native	33 (1.7%)
Asian	286 (14.3%)
Hispanic or Latino	168 (8.4%)
Native Hawaiian or other Pacific Islands	27 (1.4%)
White	1,222 (60.1%)
Work location	
Nursing home (community living center)	294 (14.7%)
Emergency medicine (urgent care, emergency department)	277 (13.8%)
Acute care inpatient (other than ICU)	631 (31.5%)
Intensive care unit - critical care	403 (20.1%)
Other work locations	399 (19.9%)

#### Quantitative analysis

We calculated unadjusted relative risks and chi-square tests to describe how all survey covariates were associated with risk of positive screens on the MIES. We then fit generalized linear mixed models using the modified Poisson method to estimate adjusted relative risks, with a log-link, robust error variance, and random intercepts for each VA facility [28]. Because a central purpose of our study was to understand how modifiable workplace factors can affect risk for moral injury, we specified *a priori* multiple stages of modeling guided by our original conceptual model (Fig 1). We added covariates in stages, with the initial stages focusing on factors conceptualized as *modifiable* (e.g., workplace/organizational culture; right side of Fig 1) and subsequent stages moving toward factors conceptualized as *intrinsic* (e.g., demographics; left side of Fig 1). This approach allowed us to evaluate how the relationships between more modifiable factors and moral injury risk might vary as more intrinsic factors were added to the model. For each stage of modeling, we classified covariates into three categories: mandatory inclusion, possible inclusion, or exclusion based on observed associations (or lack thereof) in unadjusted analyses and based on qualitative interview themes (qualitative methods are explained below). Covariates were included if unadjusted analyses or qualitative interview themes suggested a potential relationship; covariates were excluded if neither unadjusted analyses nor interviews indicated a relationship. When variables did not meet *a priori* criteria for inclusion or exclusion, we prepared versions of the model with and without them and then evaluated fit using the Bayesian Information Criterion (BIC) statistic. We also used the BIC statistic to assess overall model fit at each stage of modeling, moving from more modifiable to more intrinsic variables.

Unadjusted analyses and modeling techniques were repeated with secondary outcomes to examine their relationship to the primary outcome and to all covariates. In modeling secondary outcomes, we retained the covariates derived from the moral injury model so the relative risks estimated in each model would be comparable.

Finally, to assess moral injury risk at the facility level, we stratified facilities into quartiles based on their average scores on each facility-level metric (see Table 1) over the time period when the survey was administered. (For employee satisfaction [VA AES] measures, we weighted average scores by occupation to match the occupational distribution of survey respondents for each facility.) We then examined whether the prevalence of positive screens for moral injury was associated with metric performance across the four quartiles. We repeated this analysis for all secondary outcomes.

### Qualitative methods

#### Qualitative participants & procedures

The survey questionnaire included an optional follow-up form in which respondents could indicate their willingness to participate in the qualitative phase of the study. The follow-up form was recorded separately from survey responses but forms were auto-sorted into separate batches based on participant responses to the MIES. Interview invitations were then sent in small batches to respondents who screened as at risk for moral injury, with purposive inclusion of all participating clinical occupations and all participating facilities. Invitations were sent until preliminary analysis suggested that interviews had reached saturation. Ultimately, 46 of 413 volunteers were interviewed. The final sample included 29 nurses, 8 physicians, 3 respiratory therapists, and 6 other clinicians.

Interviews lasted approximately 60 minutes and were conducted by telephone by research analysts with training and experience in qualitative methods, supervised by the principal investigator. Interviews were audio-recorded with permission of participants.

Interviews were guided by a semi-structured interview guide. The interview guide was organized by *a priori* domains aligned with the conceptual model, including what specific conflicts they experienced, what could/should have been done differently, what was done well, and protective factors/prevention strategies at the individual, unit, and organizational levels.

#### Qualitative analysis

Interviews were analyzed in three phases. In phase one, interviewers completed a free-form memo summarizing central takeaways immediately after each interview. Memos highlighted observations pertinent to risk for moral injury. Phase two used a template-based rapid analysis technique designed to be time- and resource-efficient and to yield results that are comparable to traditional qualitative methods [29–31]. Guided by interview audio-recordings, qualitative team members prepared structured summaries of each interview using a template organized by topical areas drawn from the interview guide. This entailed summarizing participant responses for each domain and populating relevant quotations into the analysis template. To ensure consistency among analysts, more than one team member analyzed a subset of completed interviews (n = 17, or 37%). The second analyst reviewed the original templated summary and added notes documenting any questions, observations, or conflicting interpretations. Regular team meetings and written correspondence allowed the team to discuss questions, identify differences in interpretation, and achieve consensus in analytic approach. The final phase of analysis involved identification and refinement of themes through an iterative, collaborative process that combined inductive and deductive analysis. Team members began by carefully reading all templates. A lead analyst then mapped preliminary themes and supporting evidence onto a spreadsheet-based matrix pre-organized by interview domain. All qualitative team members then collaborated to review, refine, and finalize the matrix.

### Mixed-methods integration

**Fig 2:**
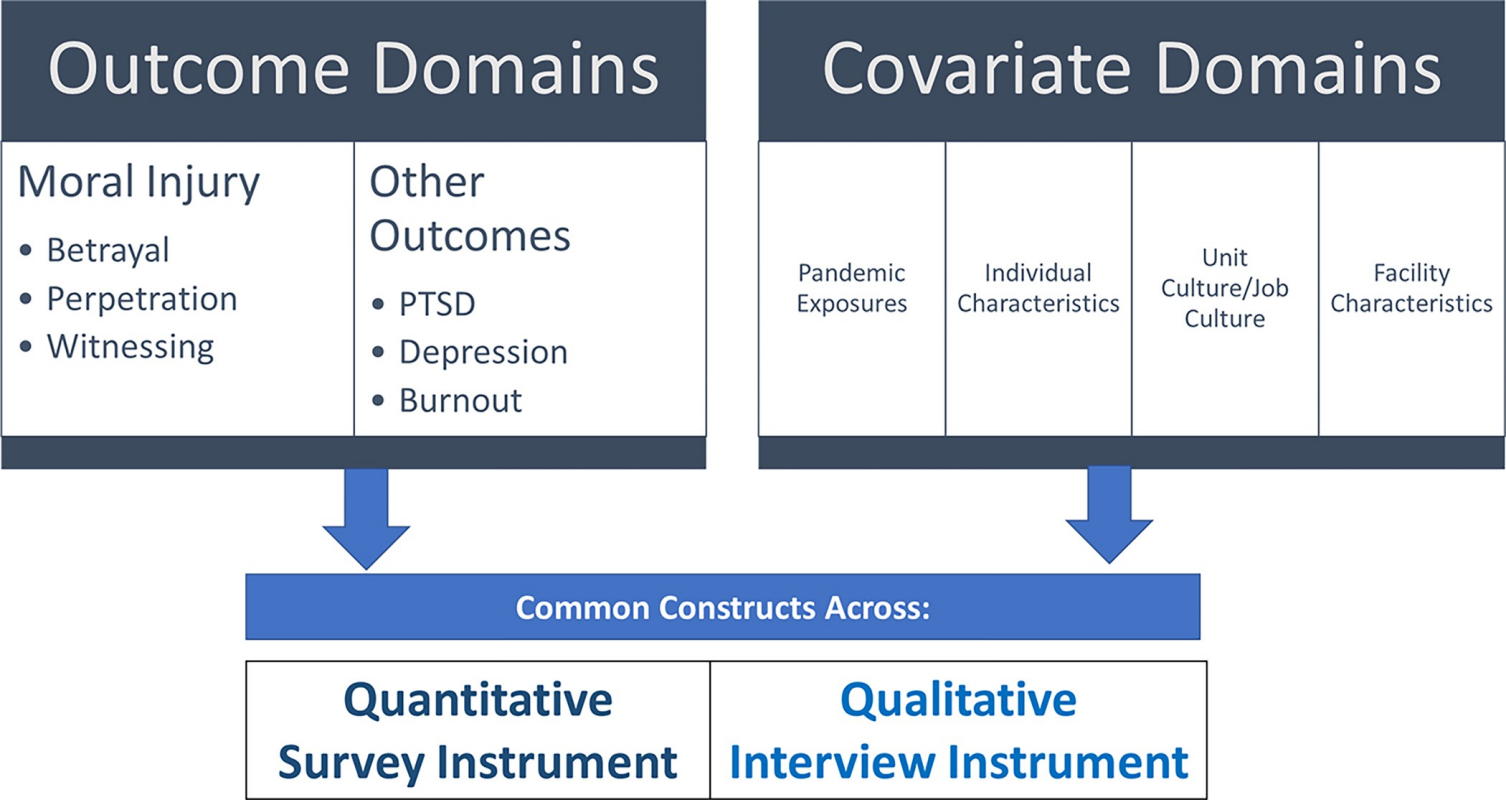
Alignment of qualitative and quantitative methods.

**Fig 3:**
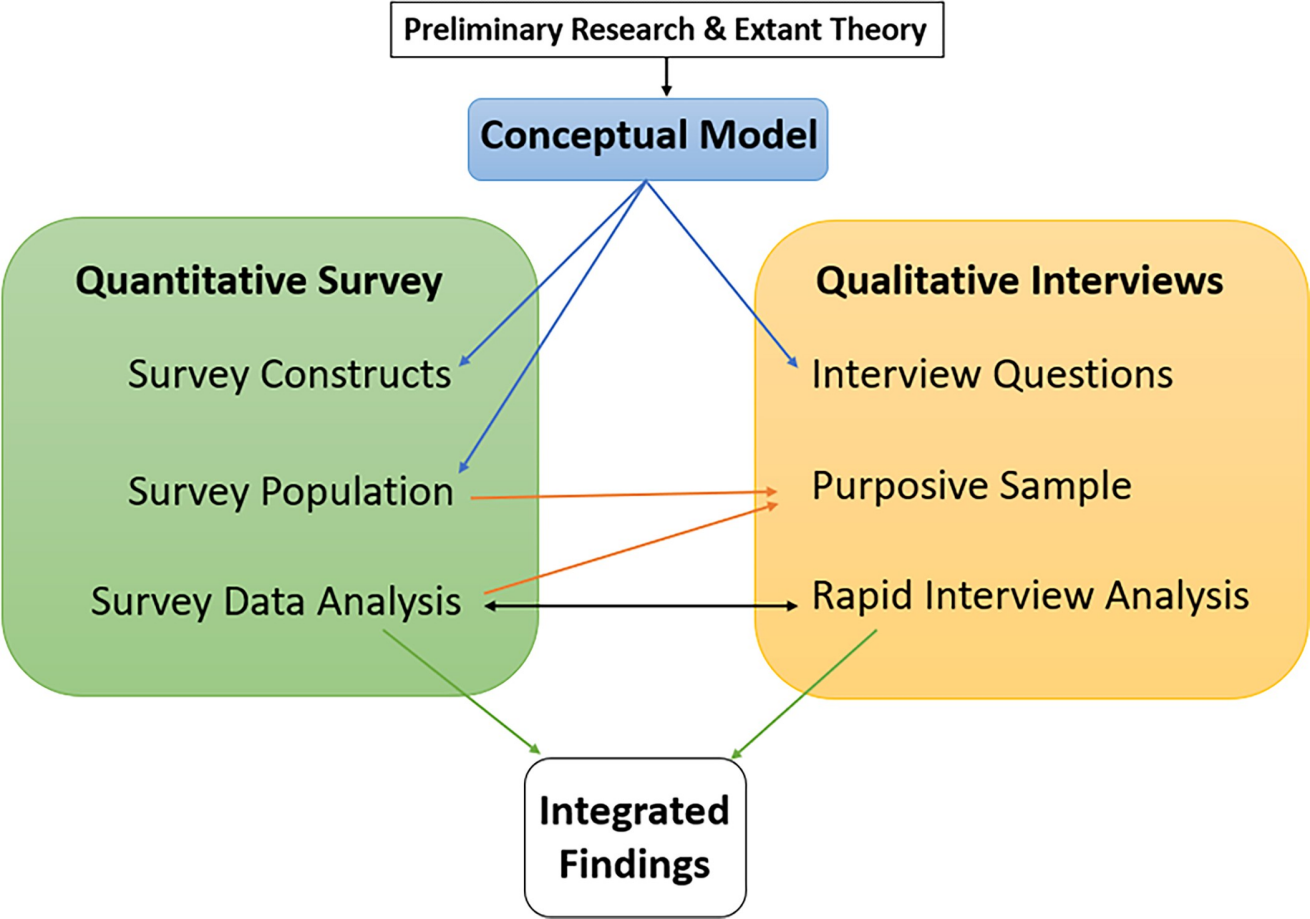
Mixed methods integration.

Qualitative and quantitative methods were integrated in several ways. In the pre-data collection phase, our initial conceptual model specified outcomes and covariates of interest, guiding the development of aligned quantitative and qualitative instruments (see Fig 2). During the data collection phase, administration of the survey prior to interviews allowed purposive sampling of clinicians who screened positive on the MIES for interview invitations, facilitating a deeper dive into the experiences of those most likely to be impacted by moral injury. During the analysis phase, preliminary interview themes derived through rapid qualitative analysis influenced ongoing quantitative analysis, informing the selection of covariates to include in the modeling process and the order in which to include them. Finally, in the concluding phase of analysis, we examined whether and how each qualitative theme related to each significant quantitative finding, preparing crosswalk tables to identify areas where qualitative data supported, expanded, contextualized, or challenged quantitative data (See Fig 3).

### Ethics statement

This study was approved by the IRB at the University of California, San Francisco and the Research and Development Committee at San Francisco Veterans Affairs Health Care System. Participants recruited for the survey received study information, a description of potential risks and benefits, and a statement that participation was voluntary and could be stopped at any time. To preserve survey participant anonymity, written consent was not obtained; instead, consent was implied by clicking the link to proceed with the survey. Verbal informed consent was obtained via telephone for all qualitative interview participants. Recruitment started on 12/10/2021 and ended on 3/26/2022.

## Results

Findings are summarized for each of the three primary research questions: (A) “Which healthcare workers and settings are at higher risk for moral injury?”, (B) “What experiences or events contribute to moral injury?”, and (C) “What workplace conditions make moral injury more or less likely?”. Section A summarizes survey and administrative data findings. Sections B and C focus primarily on qualitative interview themes. Following the description of each theme in Sections B and C, closely related quantitative findings are also highlighted to support an integrated understanding of potential contributors to moral injury in the healthcare workplace. Findings related to secondary outcomes are presented in a final, separate section entitled, “Posttraumatic Stress, Depression, and Burnout.”

### A. Which healthcare workers and settings are at higher risk for moral injury?

Descriptive statistics and unadjusted relative risks for survey covariates are presented in Table 3. Overall, 39% (773) of workers met our threshold for positive MIES screen and were therefore considered at risk for moral injury. This included 41% (656) of all workers from targeted work locations hypothesized to be at higher risk and 29% (117) from other work locations (e.g., outpatient units). Across work locations, 30% (594) felt betrayed by healthcare leaders, coworkers, or others (at risk for betrayal-based moral injury), 22% (441) were distressed by witnessing others’ unethical actions (at risk for witnessing-based moral injury), and 10% (194) felt they committed unethical actions or failed to take an ethical action they should have taken (at risk for perpetration-based moral injury).

**Table 3:** Moral injury—unadjusted analyses.

	Total	0 MI Domains		1+ MI Domain			
	(N = 2,004)	(N = 1,231)		(N = 733)			
	N	N	(%)	N	(%)	RR (95% CI)	*p*
Management health & safety support							
Strongly agree	350	302	(86.3%)	48	(13.7%)	1.00 (reference)	
Agree	605	452	(74.7%)	153	(25.3%)	1.84 (1.37, 2.48)	<0.001
Neutral	461	290	(62.9%)	171	(37.1%)	2.70 (2.03, 3.61)	<0.001
Disagree	368	137	(37.2%)	231	(62.8%)	4.58 (3.48, 6.02)	<0.001
Strongly disagree	220	50	(22.7%)	170	(77.3%)	5.63 (4.29, 7.40)	<0.001
Supervisor support							
Helpful	1,233	890	(72.2%)	343	(27.8%)	1.00 (reference)	
Not helpful	771	341	(44.2%)	430	(55.8%)	2.00 (1.80, 2.24)	<0.001
Coworker support							
Can be relied on	1,471	980	(66.6%)	491	(33.4%)	1.00 (reference)	
Can not be relied on	533	251	(47.1%)	282	(52.9%)	1.59 (1.42, 1.77)	<0.001
Coworker health climate							
Coworkers would support	1,204	843	(70.0%)	361	(30.0%)	1.00 (reference)	
Coworkers would not support	796	385	(48.4%)	411	(51.6%)	1.72 (1.54, 1.92)	<0.001
Decision empowerment							
Neutral to strongly agree	1,490	1,011	(67.9%)	479	(32.1%)	1.00 (reference)	
Disagree	358	173	(48.3%)	185	(51.7%)	1.61 (1.42, 1.82)	<0.001
Strongly disagree	156	47	(30.1%)	109	(69.9%)	2.17 (1.91, 2.47)	<0.001
Skill discretion							
Job requires creativity	1,243	801	(64.4%)	442	(35.6%)	1.00 (reference)	
Job does not require creativity	751	421	(56.1%)	330	(43.9%)	1.24 (1.11, 1.38)	<0.001
Colleague deaths							
Never	1,402	917	(65.4%)	485	(34.6%)	1.00 (reference)	
One or more times	593	313	(52.8%)	280	(47.2%)	1.36 (1.22, 1.53)	<0.001
Patient deaths							
Never	529	394	(74.5%)	135	(25.5%)	1.00 (reference)	
1-2 times	419	289	(69.0%)	130	(31.0%)	1.22 (0.99, 1.49)	0.060
3 or more times	1,056	548	(51.9%)	508	(48.1%)	1.89 (1.61, 2.21)	<0.001
Denial of Family Visits							
0–4 times	1,186	833	(70.2%)	353	(29.8%)	1.00 (reference)	
5 or more times	818	398	(48.7%)	420	(51.3%)	1.73 (1.55, 1.93)	<0.001
Lack of Supplies							
Never	470	392	(83.4%)	78	(16.6%)	1.00 (reference)	
For less than a month	453	320	(70.6%)	133	(29.4%)	1.77 (1.38, 2.27)	<0.001
For 1-2 months	393	242	(61.6%)	151	(38.4%)	2.32 (1.82, 2.94)	<0.001
For 3 or more months	688	277	(40.3%)	411	(59.7%)	3.60 (2.91, 4.45)	<0.001
Short-staffing							
2 Months or Less	754	608	(80.6%)	146	(19.4%)	1.00 (reference)	
3 Months or Longer	1,250	623	(49.8%)	627	(50.2%)	2.59 (2.22, 3.03)	<0.001
Family hospitalizations							
Never	1,768	1,103	(62.4%)	665	(37.6%)	1.00 (reference)	
One or More Times	236	128	(54.2%)	108	(45.8%)	1.22 (1.05, 1.42)	0.011
Family deaths							
Never	1,931	1,191	(61.7%)	740	(38.3%)	1.00 (reference)	
One or more times	63	34	(54.0%)	29	(46.0%)	1.20 (0.91, 1.58)	0.190
Work-family conflict							
Never to sometimes	918	728	(79.3%)	190	(20.7%)	1.00 (reference)	
Often or most of the time	1,086	503	(46.3%)	583	(53.7%)	2.59 (2.26, 2.98)	<0.001
Age							
60 years or older	279	212	(76.0%)	67	(24.0%)	1.00 (reference)	
50-59	591	388	(65.7%)	203	(34.3%)	1.43 (1.13, 1.81)	0.003
40-49	597	361	(60.5%)	236	(39.5%)	1.65 (1.31, 2.07)	<0.001
30-39	432	221	(51.2%)	211	(48.8%)	2.03 (1.62, 2.56)	<0.001
29 and under	105	49	(46.7%)	56	(53.3%)	2.22 (1.69, 2.92)	<0.001
Occupation							
Registered nurse	1,103	626	(56.8%)	477	(43.2%)	1.00 (reference)	
LPN/LVN or nursing asst.	341	226	(66.3%)	115	(33.7%)	0.78 (0.66, 0.92)	0.003
Nurse practitioner or physician asst.	65	40	(61.5%)	25	(38.5%)	0.89 (0.65, 1.22)	0.465
Physician	206	142	(68.9%)	64	(31.1%)	0.72 (0.58, 0.89)	0.003
Psychologist or social worker	42	33	(78.6%)	9	(21.4%)	0.50 (0.28, 0.89)	0.018
Other hands-on patient care	171	108	(63.2%)	63	(36.8%)	0.85 (0.69, 1.05)	0.131
Admin in clinical area, housekeeping, other	76	56	(73.7%)	20	(26.3%)	0.61 (0.42, 0.89)	0.011
Supervisory status							
Non-supervisor	1,806	1,085	(60.1%)	721	(39.9%)	1.00 (reference)	
Supervisor	198	146	(73.7%)	52	(26.3%)	0.66 (0.52, 0.84)	0.001
Gender							
Man	481	303	(63.0%)	178	(37.0%)	1.00 (reference)	
Woman, Other, Non-Binary	1,523	928	(60.9%)	595	(39.1%)	1.06 (0.92, 1.21)	0.424
Race and ethnic minority							
White	1,206	710	(58.9%)	496	(41.1%)	1.00 (reference)	
Other race/ethnic minority	798	521	(65.3%)	277	(34.7%)	0.84 (0.75, 0.95)	0.004
Work location							
Community living center	294	200	(68.0%)	94	(32.0%)	1.00 (reference)	
Emergency medicine	277	161	(58.1%)	116	(41.9%)	1.31 (1.05, 1.63)	0.015
Acute care inpatient (other than ICU)	631	387	(61.3%)	244	(38.7%)	1.21 (1.00, 1.47)	0.054
Intensive care unit–critical care	403	201	(49.9%)	202	(50.1%)	1.57 (1.29, 1.90)	<0.001
Other locations	399	282	(70.7%)	117	(29.3%)	0.92 (0.73, 1.15)	0.453

Registered nurses (RNs) screened positive on the MIES at higher rates than other occupations, with 43% (477) at risk for moral injury compared to 39% (25) of nurse practitioners and physicians’ assistants, 37% (63) of respiratory therapists and hands-on care providers not otherwise specified, 34% (115) of LPNs, LVNs, and nursing assistants, 31% (64) of physicians, 26% (20) of administrative and clerical workers, and 21% (9) of psychologists and social workers.

Moral injury risk was significantly associated with age, with a stepwise linear decrease in moral injury risk with each increase in age category: 53% (56) of the youngest workers screened positive on the MIES compared to 24% (67) of the oldest workers. Relative to workers over 60, workers under 29 were more than twice as likely to be at risk for moral injury in unadjusted analyses (RR = 2.22 [1.69- 2.92] p<0.001).

The generalized linear mixed models for moral injury are presented in Table 4. Significant covariates retained in the final model included management health and safety support, supervisor support, coworker support, decision empowerment, denial of family visits, short-staffing, and work-family conflict. Occupation was no longer a significant predictor of moral injury risk after adjusting for other covariates. Age retained significance, but the addition of age and other intrinsic factors decreased model fit.

**Table 4:** Moral injury—adjusted analyses (Generalized linear models).

	Without Intrinsic Factors		With Intrinsic Factors	
	ARR (95% CI)	*p*	ARR (95% CI)	*p*
Management health & safety support				
Strongly agree	1.00 (reference)		1.00 (reference)	
Agree	1.50 (1.09,2.06)	0.014	1.47 (1.05,2.06)	0.026
Neutral	1.86 (1.29,2.69)	<0.001	1.81 (1.24,2.65)	0.002
Disagree	2.47 (1.72,3.54)	<0.001	2.40 (1.66,3.47)	<0.001
Strongly disagree	2.70 (1.90,3.83)	<0.001	2.61 (1.82,3.76)	<0.001
Supervisor support				
Helpful	1.00 (reference)		1.00 (reference)	
Not helpful	1.15 (1.02,1.29)	0.026	1.13 (1.01,1.28)	0.040
Coworker support				
Can be relied on	1.00 (reference)		1.00 (reference)	
Can not be relied on	1.18 (1.06,1.33)	0.004	1.19 (1.07,1.33)	0.002
Decision empowerment				
Neutral to strongly agree	1.00 (reference)		1.00 (reference)	
Disagree	1.11 (0.99,1.23)	0.062	1.12 (1.00,1.25)	0.044
Strongly disagree	1.24 (1.06,1.44)	0.006	1.28 (1.09,1.49)	0.002
Patient deaths				
Never	1.00 (reference)		1.00 (reference)	
1-2 times	1.04 (0.82,1.32)	0.759	1.03 (0.82,1.29)	0.820
3 or more times	1.28 (1.09,1.50)	0.002	1.26 (1.08,1.47)	0.004
Denial of family visits				
0–4 times	1.00 (reference)		1.00 (reference)	
5 or more times	1.21 (1.12,1.31)	<0.001	1.19 (1.10,1.30)	<0.001
Short-staffing				
2 months or less	1.00 (reference)		1.00 (reference)	
3 months or longer	1.62 (1.36,1.92)	<0.001	1.58 (1.35,1.86)	<0.001
Work-family conflict				
Never to sometimes	1.00 (reference)		1.00 (reference)	
Often or most of the time	1.59 (1.37,1.83)	<0.001	1.56 (1.35,1.81)	<0.001
Age				
29 and under			1.33 (1.01,1.74)	0.039
30-39			1.33 (1.06,1.67)	0.015
40-49			1.17 (0.96,1.42)	0.114
50-59			1.14 (0.95,1.38)	0.168
60 years or older			1.00 (reference)	
Occupation				
Registered nurse			1.00 (reference)	
LPN/LVN or nursing assistant			0.94 (0.82,1.08)	0.365
Nurse practitioner or physician assistant			1.11 (0.85,1.44)	0.444
Physician			1.06 (0.84,1.34)	0.634
Psychologist or social worker			0.62 (0.38,1.01)	0.054
Other hands-on patient care			1.00 (0.87,1.15)	0.953
Admin in clinical area, housekeeping, or other			1.02 (0.73,1.43)	0.898
Supervisor status				
Non-supervisor			1.00 (reference)	
Supervisor			1.03 (0.85,1.26)	0.750
Gender				
Man			1.00 (reference)	
Woman, other, non-binary			1.05 (0.92,1.21)	0.467
Race and ethnic minority				
White			1.00 (reference)	
Other race/ethnic minority			0.98 (0.87,1.10)	0.700
Bayesian Information Criterion (BIC)		2779.7	2862.7	

When analyses were stratified by work locations hypothesized to be high-risk versus all other locations, results were very similar. Compared to participants in high-risk locations, participants in other locations reported fewer and less severe exposures to covariate risk factors and fewer positive MIES screens; however, observed associations between covariates and moral injury risk remained consistent throughout all work locations.

A separate analysis of facility-level risk did not yield consistent relationships between moral injury risk and care quality, patient safety, patient satisfaction, or staff satisfaction (Table 5). Regarding care quality, facilities that performed more poorly on the Acute Care Standardized 30-day Mortality Ratio (SMR30) had moderately increased rates of positive MIES screen relative to facilities in the top quartile of performers (RR = 1.26, p = 0.002 for third-quartile facilities; RR = 1.29, p = 0.002 for fourth-quartile facilities). However, similar trends were not observed in other metrics assessing care quality or patient safety. Findings were similarly inconsistent for facility-level measures of employee satisfaction and patient satisfaction: either the relationships that appeared statistically significant did not yield consistent patterns across quartiles, or apparent patterns were discordant across different measures of the same construct.

**Table 5:** Moral injury—facility-level metrics, unadjusted analyses.

	Total	0 MI Domains		1+ MI Domain			
	(N = 2,004)	(N = 1,231)		(N = 733)			
	N	N	(%)	N	(%)	RR (95% CI)	Pr>|Z|
Acute care standardized 30-day mortality ratio (SMR30)							
1st quartile (0.74-)	566	375	(66.3%)	191	(33.7%)	1.00 (reference)	
2nd quartile (0.98-)	445	290	(65.2%)	155	(34.8%)	1.03 (0.87, 1.23)	0.719
3rd quartile (1.05-)	566	325	(57.4%)	241	(42.6%)	1.26 (1.09, 1.47)	0.002
4th quartile (1.20-1.59)	426	241	(56.6%)	185	(43.4%)	1.29 (1.10, 1.51)	0.002
Hospital 30-day readmission rate							
1st quartile (11.47-)	589	356	(60.4%)	233	(39.6%)	1.00 (reference)	
2nd quartile (12.22-)	486	309	(63.6%)	177	(36.4%)	0.92 (0.79, 1.07)	0.294
3rd quartile (12.92-)	561	336	(59.9%)	225	(40.1%)	1.01 (0.88, 1.17)	0.849
4th quartile (13.44-13.92)	367	230	(62.7%)	137	(37.3%)	0.94 (0.80, 1.11)	0.497
Patient safety indicator (PSI90)							
1st quartile (0.71-)	525	352	(67.0%)	173	(33.0%)	1.00 (reference)	
2nd quartile (0.82-)	508	319	(62.8%)	189	(37.2%)	1.13 (0.96, 1.33)	0.153
3rd quartile (0.92-)	609	334	(54.8%)	275	(45.2%)	1.37 (1.18, 1.59)	<0.001
4th quartile (1.10-1.42)	361	226	(62.6%)	135	(37.4%)	1.13 (0.95, 1.36)	0.171
PCMH care coordination							
4th quartile (65.59-70.20)	475	275	(57.9%)	200	(42.1%)	1.00 (reference)	
3rd quartile (61.65-)	501	324	(64.7%)	177	(35.3%)	0.84 (0.72, 0.98)	0.030
2nd quartile (58.40-)	521	321	(61.6%)	200	(38.4%)	0.91 (0.78, 1.06)	0.230
1st quartile (53.29-)	506	311	(61.5%)	195	(38.5%)	0.92 (0.79, 1.07)	0.254
HCAHPS care transition							
4th quartile (55.02-57.89)	423	256	(60.5%)	167	(39.5%)	1.00 (reference)	
3rd quartile (50.98-)	574	360	(62.7%)	214	(37.3%)	0.94 (0.81, 1.11)	0.478
2nd quartile (45.63-)	488	290	(59.4%)	198	(40.6%)	1.03 (0.88, 1.21)	0.734
1st quartile (43.48-)	518	325	(62.7%)	193	(37.3%)	0.94 (0.80, 1.11)	0.484
HCAHPS hospital overall rating							
4th quartile (75.23-79.79)	481	270	(56.1%)	211	(43.9%)	1.00 (reference)	
3rd quartile (71.58-)	478	300	(62.8%)	178	(37.2%)	0.85 (0.73, 0.99)	0.038
2nd quartile (65.07-)	527	345	(65.5%)	182	(34.5%)	0.79 (0.67, 0.92)	0.003
1st quartile (60.73-)	517	316	(61.1%)	201	(38.9%)	0.89 (0.76, 1.03)	0.110
Best places to work (AES)							
4th quartile (74.49-83.06)	523	297	(56.8%)	226	(43.2%)	1.00 (reference)	
3rd quartile (71.02-)	418	275	(65.8%)	143	(34.2%)	0.79 (0.67, 0.93)	0.006
2nd quartile (67.36-)	545	350	(64.2%)	195	(35.8%)	0.83 (0.71, 0.96)	0.013
1st quartile (63.54-)	517	309	(59.8%)	208	(40.2%)	0.93 (0.81, 1.08)	0.332
Organization satisfaction (AES)							
4th quartile (3.90-4.12)	523	297	(56.8%)	226	(43.2%)	1.00 (reference)	
3rd quartile (3.73-)	418	275	(65.8%)	143	(34.2%)	0.79 (0.67, 0.93)	0.006
2nd quartile (3.68-)	560	354	(63.2%)	206	(36.8%)	0.85 (0.74, 0.99)	0.031
1st quartile (3.51-)	502	305	(60.8%)	197	(39.2%)	0.91 (0.78, 1.05)	0.197
Registered nurse turnover (facility total loss rate)							
1st quartile (8.79-)	519	342	(65.9%)	177	(34.1%)	1.00 (reference)	
2nd quartile (11.58-)	464	290	(62.5%)	174	(37.5%)	1.10 (0.93, 1.30)	0.267
3rd quartile (13.15-)	550	321	(58.4%)	229	(41.6%)	1.22 (1.05, 1.43)	0.012
4th quartile (14.02-20.67)	470	278	(59.1%)	192	(40.9%)	1.20 (1.02, 1.41)	0.029

Of all facility-level metrics, the SMR30 yielded the strongest indication of a potential relationship to moral injury. Therefore, we conducted a sensitivity analysis to determine how its addition to the moral injury model would impact fit. We found that it decreased model fit.

### B. What experiences or events contribute to moral injury?

#### Institutional betrayal

Interviewed workers described feeling betrayed and abandoned by healthcare leaders and managers as an important risk factor for moral injury. This occurred when leaders’ decisions, actions, and/or inactions were seen as causing harm to the participants, their coworkers, and/or their patients. Examples included leaders’ failing to listen to and learn about the resource and staffing needs of frontline staff, failing to be visible to or present with frontline staff during the pandemic, or establishing policies and procedures that impacted working conditions or patient care without adequately consulting frontline staff.

This betrayal was felt most acutely when it negatively impacted patient care. In the words of an ICU RN, “I was desperately trying to do what I felt was right and best for my patients and not only did I feel unheard and unvalued by my leadership, but I felt handicapped by them, and I think that was particularly difficult… It felt like… my coworkers and I were betrayed and abandoned and left to [do] more with less and less.” Another inpatient RN said, “We felt like we were left to our own devices…. We got a lot of lip service and no actual action… It’s demoralizing and it’s disheartening.”

This qualitative theme aligns with survey data, where 30% of respondents endorsed two or more items in the betrayal domain of the MIES. Notably, one of the strongest predictors of moral injury risk was perceived management support for workplace health and safety. Surveyed workers who strongly disagreed with the statement that “management considers workplace health and safety to be important” were greater than five times more likely to be at risk for moral injury compared to those who strongly agreed. In the final model, the adjusted relative risk (the contribution of this covariate over and above the contribution of all others in the model) was 2.70 (p<0.001).

#### Values-policy conflict

Interviewed workers described following hospital policies that violated their own values as a significant contributor to moral injury. The most common example of this was enforcing rules requiring isolation of critically ill patients from loved ones or long-term isolation of nursing home residents from families. For example, an inpatient nurse practitioner found it extremely distressing when she was unable to allow a dying patient time with their young child: “We had a family member who had a 4-year old. This patient was dying and they wanted to see their baby one last time… They couldn’t see him, was the bottom line, they couldn’t see him.” Similarly, an ICU RN described the moral distress created by pandemic-era visitation restrictions: “We were taking care of them and they were dying without their family…. After they’re dead, they’re dead, and those policies I felt were wrong… That family member, if they want to suit up and wear the same protective gear that we have to wear… they should be given that choice.”

Survey data showed a significant correlation between moral injury risk and denial of family visits to critically ill patients. Workers who denied family visits to critically ill patients more than four times were 73% more likely to be at risk for moral injury than those who did not deny family visits. In the final model, the adjusted relative risk was 1.21 (p<0.001).

#### Compromised care quality

Interviewed workers experienced intense moral distress when they felt unable to provide the standard of care that patients deserved due to inadequate staffing and inadequate time to spend with each patient, or due to lack of needed supplies and resources (e.g., personal protective equipment). An emergency room nurse described feeling distress at “watching these people just be stranded or stuck in an emergency department” when the facility ran out of beds: “sometimes, just to get them moved, people would place them in the wrong level of care.” An ICU nurse felt intense moral conflict due to short-staffing in her department that prevented her from monitoring intubated COVID-19 patients with the frequency she felt was required: “We were expected to monitor those patients like once every four hours. And that felt extremely uncomfortable… You can’t appropriately paralyze somebody and not check them in four hours… They couldn’t protect themselves, you couldn’t protect them, that just wasn’t safe.”

This qualitative theme aligns with survey findings, which showed a significant correlation between moral injury risk and duration of short-staffing during the pandemic. Workers who reported that their team lacked personnel required for patient care for three months or longer were more than twice as likely to be at risk for moral injury compared to those who reported more transient short-staffing. In the final model, the adjusted relative risk was 1.62 (p<0.001).

#### Powerlessness to mitigate suffering

Interviewed workers described feeling powerless to help dying patients as a significant contributor to moral injury: "We’re losing people left and right, and there’s nothing we can do.” An ICU RN acknowledged “the distress of knowing with almost certainty that every patient you cared for was probably going to die…. I remember experiencing a significant amount of moral and ethical conflict.” Interviewees’ distress was particularly acute when their patients were suffering. They felt that patients were relying on them and yet they could not deliver care that would mitigate their suffering or prevent their deaths. “When the patients are asking you to save them… that’s the most distressing part,” explained another ICU RN.

This qualitative theme aligns with and helps to explain survey findings, which showed a significant correlation between moral injury risk and the deaths of patients under one’s care during the COVID-19 pandemic. Workers who cared for three or more COVID-19 patients who died were 89% more likely to be at risk for moral injury than those who did not experience a patient death from COVID-19. In the final model, the adjusted relative risk was 1.28 (p = 0.002).

Yet, interviews suggested that not all deaths were equal in the eyes of healthcare workers; some were far more distressing than others, and many interviewees described at least one patient loss that haunted them. Failure to provide patients a death with dignity or to pause and honor those who died could be especially distressing. An LVN, for example, was deeply troubled that, when “your patient dies… you just got to throw ‘em in a body bag and clean the room and there’s another one waiting in the ER for the bed.”

#### Work-family conflict

Interviewed workers felt that conflicts between their duties at work and their obligations to family and loved ones contributed to moral injury. Especially during the COVID-19 pandemic, workers feared putting their own family at risk of contagious illness. An inpatient nurse explained, "We all understood that, on the one hand, we wanted to take care of patients like we always had… One part of our mind was focused on the job…. Another part of our mind, for every nurse I know, was also worried about ’what if I bring this home’ to whoever was their special immunocompromised person … Every single person had somebody in their circle they were especially worried about bringing it home to."

Interviewed workers also reported moral distress related to neglecting the needs of loved ones because work demanded all of their time and energy. They felt they had nothing left to give at home. Another inpatient RN shared that her “parenting was severely affected… I just wanted to be in my room when I wasn’t at work… I’m just exhausted and mentally not there.”

This qualitative theme aligns with survey findings, which showed a significant correlation between moral injury risk and work-family conflict. Workers who reported experiencing conflict most or all of the time were more than twice as likely to be at risk for moral injury than those who reported less frequent work-family conflict. In the final model, the adjusted relative risk was 1.59 (p<0.001).

#### Futile care

A significant source of moral injury described by interviewees was feeling bound to deliver care that is futile and causes suffering. This happened often in the context of COVID-19 care but was relevant in other contexts as well. “It was really hard to watch painful procedures, painful care be done to people in the name of ’oh, well maybe we can get them better,’” shared an ICU RN, “The futile care was something I really struggled with.” Another ICU RN who cared for COVID-19 patients said she “felt like [we] were torturing them [patients] by proning them, then being on blood pressure medication and being intubated and sedated for more than two weeks and we knew the patient wasn’t going to be able to come off the ventilator.” The word “torture” was used by multiple interviewees in the context of delivering futile care: “At a certain point, you’re just literally torturing them and you’re a part of that. Emotionally, it was draining, still is to this day,” shared a respiratory therapist.

There were no survey questions linked with this theme, but it emerged during qualitative interviews as a novel theme and a significant contributor to moral injury.

#### Communication failures

A related source of moral injury involved high-stakes provider-family communication failures. Specifically, interviewees reported feeling distressed that patients with dire prognoses and their families were sometimes not adequately and honestly informed of those prognoses. They felt that false hope affected their treatment decisions and that healthcare providers were not always doing enough to deliver difficult-to-hear, honest information. An ICU RN described the case of a COVID-19 patient she cared for: “Those two docs, the first time they saw him, knew there’s no chance. But we, for three and a half months, we had this guy, our doc could never tell her there’s no chance… It’s just wrong, it’s torture.” Another ICU RN was similarly distressed at these communication failures: “When you go to work, you feel like you’re failing families because they’re not being cared for or told information that you would want to know.”

Again, there were no survey questions linked with this theme, but it emerged during qualitative interviews as a novel theme and a significant contributor to moral injury.

Table 6 integrates qualitative and quantitative data to summarize the types of experiences and events that contributed to moral injury.

**Table 6:** What experience or events contribute to moral injury? integrated table.

Experience/Event	Qualitative Theme	Quantitative Support		
		Survey Construct	Unadjusted RR	Adjusted RR
Institutional Betrayal	Feeling betrayed and abandoned by healthcare leaders and managers whose decisions, actions, and inaction cause harm to oneself, one’s coworkers, and/or one’s patients.	Management Health & Safety Support Construct *(strongly disagree relative to strongly agree)*	5.63*	2.70*
Values-Policy Conflict	Following hospital policies that violate one’s own values, such as rules requiring isolation of critically ill patients from loved ones, or long-term isolation of community living center residents from their families.	Denying Family Visits to Critically Ill Patients *(5 or more denials relative to less than 5)*	1.73*	1.21*
Compromised Care Quality	Being unable to provide the standard of care that patients deserve due to inadequate staffing and inadequate time to spend with each patient, or lack of supplies and other resources.	Short-Staffing *(3+ months of short-staffing relative to shorter duration)*	2.59*	1.62*
Powerlessness to Mitigate Suffering	Feeling powerless to help patients who are suffering or dying, and who are relying on you to help them.	Patient Deaths from COVID-19 *(3 or more deaths relative to none)*	1.89*	1.28**
Work-Family Conflict	Experiencing work-family conflict—for example, putting one’s own family at risk of contagious illness, or neglecting the needs of loved ones because work is demanding all of one’s time and energy.	Work-Family Conflict *(all or most of the time relative to less frequent)*	2.59*	1.59*
Futile Care	Feeling bound to deliver care that is futile and causes suffering.	N/A		
Communication Failures	Feeling that patients with dire prognoses and their families are not adequately and honestly informed, and that false hope affects their treatment decisions.	N/A		

*p<0.001

**p = 0.002.

### C. What workplace conditions make moral injury more or less likely?

#### Community versus isolation

Across interviews, participants felt that the presence of a supportive workplace community provided some protection against moral injury. When distressing events were not faced alone and responsibility was meaningfully shared, workers felt that morally distressing events were less likely to happen or less likely to result in lasting moral injury. By contrast, there was little buffer against moral injury where there was lack of community, connection, and belonging – where, instead, individuals felt alienated from others and burdened by responsibility. A morally injured inpatient RN described “feeling like left-overs… Nobody wants to come to us, but we have to show up every time and do the job.” Another RN lamented that “nobody came to talk to us to make sure that we were mentally okay.”

This qualitative theme aligns with survey findings, which showed a significant correlation between moral injury risk and perceived coworker support. Workers who reported that their co-workers could not be relied on to help were 59% more likely to be at risk for moral injury compared to those who reported that they could rely on their co-workers. In the adjusted model, the relative risk was 1.18 (p = 0.004).

#### Responsible versus absent leaders

Interviewees felt that moral injury was less likely when leaders, managers, and decision-makers were present and in communication with those on the frontlines. They emphasized the importance of meaningful communication in both directions so frontline workers feel genuinely heard and appreciated. When leaders, managers and decision-makers seemed disengaged from the frontlines, interviewees described feeling greater and more frequent moral distress. This was particularly true when leaders lacked understanding of the experiences of workers and made important decisions without listening carefully for their input. Unfortunately, this was a common experience. One ICU RN shared, "My manager was not really there. She was not involved, she did not glove up, put PPE on, and actually work. She has not actually stepped into a COVID room but yet she’s representing her staff and she has no idea what she’s asking her staff to do.” Similarly, an ER physician explained that his feelings of betrayal would have been eased "if they [hospital leaders] would appear to understand how hard this has been for us, or acknowledge that…but, like I said, we don’t see them.”

In alignment with this qualitative theme, survey data showed a significant correlation between moral injury risk and perceived supervisor support. Workers who disagreed that their supervisors were helpful in getting the job done were twice as likely to be at risk for moral injury compared to those who agreed. In the final model, the adjusted relative risk was 1.15 (p = 0.026).

#### Empowered versus powerless workers

When interviewees saw that it was possible to make meaningful changes to their workplace and to address the factors that caused moral distress, they experienced this as a mitigating factor that could reduce moral injury risk. By contrast, workers’ distress and frustration were amplified when they lacked agency to improve the situation or to do things differently in the future. For example, a respiratory therapist explained that his own distress would have been alleviated “if management had only asked… ‘what could be learned from this, what could be changed?’” But this did not happen.

This qualitative theme helps to explain survey findings, which showed a significant correlation between moral injury risk and perceived empowerment in the workplace. Workers who strongly disagreed they had the authority to make decisions that impacted them at work were more than twice as likely to be at risk for moral injury than those who agreed or responded neutrally. In the final model, the adjusted relative risk was 1.24 (p = 0.006).

#### Processing versus suppressing

Across interviews, the presence of meaningful breaks that allowed for reflection and recovery was identified as a factor that could provide some protection against moral injury. Workers felt that morally distressing events were less likely to result in lasting moral injury when there was time to pause and opportunity to process and discuss these events. Morally injured workers described feeling compelled to ignore or suppress their feelings so they could continue to operate under high-stress conditions. For them, this could feel unrealistic and inhumane. One ICU RN shared, "I took care of [Tom] for four weeks. I took care of [Chris] for two weeks. I took care of [Steve] for three weeks. I took care of [Andy] for four. You don’t get to take care of these people every day and then put them in body bags and it doesn’t impact you.” Those losses, she felt, had to be acknowledged and grieved.

There were no survey questions linked with this theme, but it emerged during qualitative interviews as a novel theme and a notable factor impacting moral injury risk.

#### Shared risk versus uneven burdens

Although different jobs carry different risks and burdens, interviewed workers appreciated it when the organization made an effort to share the heaviest burdens, meaningfully acknowledge workers who carried more than their share of burden, and take action to make things more balanced when there were pronounced inequities. By contrast, interviewees felt that moral injury was more likely when certain workers were consistently asked to bear a disproportionate share of workplace risk and burden over a long period of time. A common example was the routine delegation of inpatient COVID-19 care to a very small number of nurses in order to protect other employees from any contact with COVID-19 inpatients. “We bore the brunt of this pandemic,” shared an exhausted inpatient nurse. Workers also felt frustrated and betrayed when their sacrifices were acknowledged in only superficial ways and there were no meaningful efforts to ensure a fairer distribution of risk and burden. An emergency room nurse described feeling “abandonment by higher up people.”

There were no survey questions linked with this theme, but it emerged during qualitative interviews as a novel theme and a notable factor impacting moral injury risk.

Table 7 integrates qualitative and quantitative data to summarize workplace conditions affecting moral injury risk.

**Table 7:** What workplace conditions make moral injury more or less likely? integrated table.

Context	Moral injury is less likely when…	Moral injury is more likely when…	Quantitative Support		
			Survey Construct	Unadjusted RR	Adjusted RR
Community *versus* Isolation	Community support is present; distressing events are not faced alone, and responsibility is meaningfully shared.	There is little sense of connection, belonging, or community; instead, individuals feel alienated from others and burdened by responsibility.	Coworker Support *(co-workers cannot be relied on relative to can be relied on)*	1.59*	1.18**
Responsible *versus* Absent Leaders	Leaders, managers, and decision-makers are present and in communication with those on the frontlines. There is meaningful communication in both directions so frontline workers feel genuinely heard and appreciated.	Leaders, managers and decision-makers seem absent or disengaged from the frontlines. They lack understanding of the experiences of workers and make important decisions without listening carefully for their input.	Supervisor Support *(disagree that supervisors are helpful relative to agree)*	2.00*	1.15***
Empowered *versus* Powerless	It is possible to make meaningful changes to the situation or environment and address the factors that caused moral distress.	Those experiencing moral distress feel like they have no real agency to change or improve the situation, or to do things differently in the future.	Decision Empowerment *(strongly disagree relative to agree that they have authority to make job decisions)*	2.17*	1.24**
Processing *versus* Suppressing	There is time and opportunity to process and discuss distressing events; there are breaks that allow reflection and recovery.	Distressing events are not processed or discussed; there is no room for a pause. People must try to ignore or suppress their feelings so they can continue to operate under high-stress conditions.	N/A		
Shared Risk *versus* Uneven Burdens	Although different jobs carry different risks and burdens, there is an effort to share the heaviest burdens, meaningfully acknowledge workers who carry more than their share, and take action to make things more balanced when there are pronounced inequities.	Certain workers are consistently asked to bear a disproportionate share of workplace risk and burden over a long period of time. Their sacrifices are acknowledged in only superficial ways and there are no meaningful efforts to ensure a fairer distribution of risk and burden.	N/A		

*p<0.001

**p<0.01

***p = 0.026.

### Posttraumatic stress, depression, and burnout

As reported in Table 8, 41% (818) of surveyed workers screened positive for posttraumatic stress risk, 27% (545) for depression risk, and 25% (503) for persistent burnout risk. Compared to workers *not* at risk for moral injury, workers who were at risk for moral injury were more than twice as likely to be at risk for posttraumatic stress (RR = 2.10 [1.89-2.33), p=<0.001) and depression (RR = 2.08 [1.80-2.40], p<0.001), and more than three times as likely to screen positive for persistent burnout (RR = 3.20 [2.72, 3.77], p<0.001). Correlation coefficients between moral injury and secondary outcomes, which ranged from weak to moderate, are presented in Table 9.

**Table 8:** PTSD, depression, and burnout—unadjusted analyses.

	PTSD		Depression		Burnout	
	RR (95% CI)	*p*	RR (95% CI)	*p*	RR (95% CI)	*p*
Management health & safety support						
Strongly agree						
Agree	1.37 (1.10, 1.72)	0.006	1.36 (1.01, 1.83)	0.046	1.74 (1.17, 2.57)	0.006
Neutral	1.82 (1.46, 2.27)	<0.001	1.93 (1.44, 2.59)	<0.001	2.92 (2.00, 4.25)	<0.001
Disagree	2.65 (2.15, 3.27)	<0.001	2.58 (1.94, 3.44)	<0.001	4.57 (3.17, 6.58)	<0.001
Strongly disagree	2.64 (2.12, 3.30)	<0.001	3.27 (2.45, 4.36)	<0.001	6.58 (4.58, 9.44)	<0.001
Supervisor support						
Helpful						
Not helpful	1.43 (1.29, 1.59)	<0.001	1.51 (1.31, 1.74)	<0.001	2.35 (2.01, 2.74)	<0.001
Coworker support						
Can be relied on						
Can not be relied on	1.28 (1.15, 1.43)	<0.001	1.50 (1.30, 1.73)	<0.001	1.74 (1.49, 2.02)	<0.001
Coworker health climate						
Coworkers would support						
Coworkers would not support	1.58 (1.43, 1.76)	<0.001	1.53 (1.33, 1.76)	<0.001	1.80 (1.55, 2.09)	<0.001
Decision empowerment						
Neutral to strongly agree						
Disagree	1.31 (1.16, 1.49)	<0.001	1.66 (1.41, 1.95)	<0.001	2.28 (1.94, 2.69)	<0.001
Strongly disagree	1.44 (1.23, 1.69)	<0.001	1.94 (1.59, 2.36)	<0.001	3.00 (2.51, 3.60)	<0.001
Skill discretion						
Job requires creativity						
Job does not require creativity	1.06 (0.95, 1.18)	0.312	1.18 (1.02, 1.37)	0.024	1.31 (1.12, 1.52)	0.001
Colleague deaths						
Never						
One or more times	1.53 (1.38, 1.70)	<0.001	1.32 (1.14, 1.52)	<0.001	1.10 (0.94, 1.30)	0.226
Patient deaths						
Never						
1-2 times	1.55 (1.25, 1.91)	<0.001	0.94 (0.75, 1.18)	0.603	1.18 (0.91, 1.52)	0.215
3 or more times	2.45 (2.06, 2.91)	<0.001	1.20 (1.01, 1.43)	0.038	1.61 (1.31, 1.96)	<0.001
Denial of Family Visits						
0–4 times						
5 or more times	1.69 (1.52, 1.87)	<0.001	1.26 (1.10, 1.46)	0.001	1.47 (1.26, 1.70)	<0.001
Lack of Supplies						
Never						
For less than a month	1.63 (1.33, 1.99)	<0.001	1.36 (1.04, 1.76)	0.022	1.45 (1.06, 1.97)	0.019
For 1-2 months	1.78 (1.46, 2.17)	<0.001	1.52 (1.17, 1.97)	0.002	2.03 (1.51, 2.72)	<0.001
For 3 or more months	2.26 (1.90, 2.70)	<0.001	2.19 (1.76, 2.74)	<0.001	3.03 (2.35, 3.92)	<0.001
Short-staffing						
2 Months or Less						
3 Months or Longer	1.75 (1.54, 1.99)	<0.001	1.83 (1.54, 2.18)	<0.001	2.73 (2.21, 3.36)	<0.001
Family hospitalizations						
Never						
One or More Times	1.34 (1.17, 1.53)	<0.001	1.26 (1.03, 1.53)	0.024	1.21 (0.98, 1.50)	0.078
Family deaths						
Never						
One or more times	1.50 (1.22, 1.84)	<0.001	1.36 (0.98, 1.91)	0.067	1.14 (0.77, 1.70)	0.516
Work-family conflict						
Never to sometimes						
Often or most of the time	2.50 (2.19, 2.84)	<0.001	3.71 (3.04, 4.52)	<0.001	4.98 (3.95, 6.29)	<0.001
Age						
60 years or older						
50-59	1.56 (1.25, 1.95)	<0.001	1.35 (1.03, 1.76)	0.028	1.64 (1.18, 2.28)	0.004
40-49	1.70 (1.36, 2.12)	<0.001	1.32 (1.01, 1.73)	0.039	1.82 (1.31, 2.53)	<0.001
30-39	1.76 (1.41, 2.21)	<0.001	1.48 (1.13, 1.94)	0.005	2.46 (1.78, 3.41)	<0.001
29 and under	2.25 (1.73, 2.91)	<0.001	1.49 (1.03, 2.15)	0.036	2.80 (1.91, 4.10)	<0.001
Occupation						
Registered nurse						
LPN/LVN or nursing asst.	1.00 (0.88, 1.15)	0.976	1.05 (0.87, 1.26)	0.610	0.81 (0.65, 1.01)	0.057
Nurse practitioner or physician asst.	0.86 (0.63, 1.18)	0.342	0.80 (0.51, 1.26)	0.342	0.92 (0.61, 1.41)	0.711
Physician	0.54 (0.42, 0.70)	<0.001	0.39 (0.26, 0.58)	<0.001	0.60 (0.44, 0.82)	0.002
Psychologist or social worker	0.53 (0.31, 0.92)	0.023	0.99 (0.61, 1.62)	0.976	0.84 (0.49, 1.46)	0.542
Other hands-on patient care	0.85 (0.69, 1.04)	0.112	0.96 (0.74, 1.24)	0.734	0.74 (0.55, 1.01)	0.057
Admin in clinical area, housekeeping, other	0.62 (0.43, 0.89)	0.010	1.30 (0.95, 1.77)	0.097	0.70 (0.44, 1.11)	0.129
Supervisory status						
Non-supervisor						
Supervisor	0.67 (0.53, 0.84)	0.001	0.74 (0.56, 0.98)	0.038	0.94 (0.72, 1.22)	0.638
Gender						
Man						
Woman, other, non-binary	1.05 (0.93, 1.19)	0.441	0.92 (0.78, 1.09)	0.342	0.96 (0.80, 1.14)	0.610
Race and ethnic minority						
White						
Other race/ethnic minority	1.09 (0.98, 1.21)	0.107	0.91 (0.79, 1.06)	0.222	0.77 (0.65, 0.90)	0.001
Work location						
Community living center						
Emergency medicine	1.35 (1.09, 1.68)	0.005	1.19 (0.91, 1.56)	0.211	1.40 (1.04, 1.89)	0.026
Acute care inpatient (other than ICU)	1.28 (1.06, 1.55)	0.012	0.97 (0.76, 1.24)	0.834	1.27 (0.97, 1.66)	0.077
Intensive care unit–critical care	1.80 (1.49, 2.17)	<0.001	1.34 (1.05, 1.71)	0.018	1.53 (1.16, 2.01)	0.002
Other locations	0.89 (0.71, 1.12)	0.332	1.10 (0.85, 1.43)	0.447	1.10 (0.82, 1.48)	0.522

**Table 9:** Correlation table for outcome measures, Spearman’s Rho.

	Any MI	PTSD	Depression	Burnout	MI Perpetration	MI Betrayal	MI Witnessing
Any MI	1.00						
PTSD	0.31	1.00					
Depression	0.22	0.27	1.00				
Burnout	0.34	0.24	0.40	1.00			
MI Perpetration	0.41	0.20	0.15	0.17	1.00		
MI Betrayal	0.82	0.26	0.23	0.32	0.30	1.00	
MI Witnessing	0.67	0.27	0.16	0.25	0.40	0.41	1.00

In GLMs, relationships between secondary outcomes and covariates of interest were similar to those identified for moral injury but did not always reach statistical significance (Tables 10 and 11). After adjustments, management health and safety support, short-staffing, and work-family conflict were significant predictors of posttraumatic stress, depression, and burnout. Coworker support and decision empowerment were significant predictors of positive depression and burnout screens. Patient deaths and denial of family visits were significant predictors of positive posttraumatic stress screen.

**Table 10:** PTSD, depression, and burnout—adjusted analyses without intrinsic factors.

	PTSD		Depression		Burnout	
	ARR (95% CI)	*p*	ARR (95% CI)	*p*	ARR (95% CI)	*p*
Management health & safety support						
Strongly agree	1.00 (reference)		1.00 (reference)		1.00 (reference)	
Agree	1.12 (0.93,1.36)	0.229	1.06 (0.74,1.52)	0.756	1.23 (0.84,1.81)	0.294
Neutral	1.38 (1.13,1.70)	0.002	1.30 (0.86,1.96)	0.207	1.59 (1.11,2.28)	0.012
Disagree	1.65 (1.29,2.12)	<0.001	1.39 (1.02,1.91)	0.040	1.77 (1.20,2.61)	0.004
Strongly disagree	1.58 (1.26,1.98)	<0.001	1.65 (1.06,2.57)	0.026	2.17 (1.41,3.35)	<0.001
Supervisor support						
Helpful	1.00 (reference)		1.00 (reference)		1.00 (reference)	
Not helpful	0.97 (0.88,1.08)	0.636	0.90 (0.77,1.04)	0.153	1.18 (0.99,1.41)	0.062
Coworker support						
Can be relied on	1.00 (reference)		1.00 (reference)		1.00 (reference)	
Can not be relied on	1.08 (0.99,1.17)	0.082	1.19 (1.08,1.32)	<0.001	1.20 (1.03,1.39)	0.017
Decision empowerment						
Neutral to strongly agree	1.00 (reference)		1.00 (reference)		1.00 (reference)	
Disagree	1.04 (0.93,1.16)	0.491	1.28 (1.12,1.45)	<0.001	1.53 (1.38,1.70)	<0.001
Strongly disagree	1.02 (0.91,1.14)	0.763	1.31 (1.07,1.59)	0.009	1.63 (1.36,1.95)	<0.001
Patient deaths						
Never	1.00 (reference)		1.00 (reference)		1.00 (reference)	
1-2 times	1.39 (1.17,1.64)	<0.001	0.81 (0.66,1.00)	0.049	0.97 (0.87,1.09)	0.615
3 or more times	1.83 (1.63,2.06)	<0.001	0.89 (0.75,1.06)	0.186	1.09 (0.89,1.33)	0.397
Denial of family visits						
0–4 times	1.00 (reference)		1.00 (reference)		1.00 (reference)	
5 or more times	1.15 (1.01,1.32)	0.042	0.96 (0.85,1.09)	0.527	0.99 (0.82,1.20)	0.942
Short-staffing						
2 months or less	1.00 (reference)		1.00 (reference)		1.00 (reference)	
3 months or longer	1.18 (1.06,1.32)	0.002	1.20 (1.02,1.42)	0.026	1.50 (1.29,1.76)	<0.001
Work-family conflict						
Never to sometimes	1.00 (reference)		1.00 (reference)		1.00 (reference)	
Often or most of the time	1.84 (1.56,2.17)	<0.001	3.12 (2.19,4.46)	<0.001	3.28 (2.51,4.28)	<0.001

**Table 11:** PTSD, depression, and burnout—adjusted analyses with intrinsic factors.

	PTSD			Depression			Burnout	
	ARR (95% CI)	*p*	ARR (95% CI)		*p*	ARR (95% CI)		*P*
Management health & safety support								
Strongly agree	1.00 (reference)		1.00 (reference)			1.00 (reference)		
Agree	1.13 (0.95,1.33)	0.157	1.05 (0.73,1.49)		0.796	1.25 (0.85,1.82)		0.256
Neutral	1.31 (1.09,1.58)	0.003	1.25 (0.84,1.85)		0.280	1.61 (1.14,2.26)		0.007
Disagree	1.59 (1.27,1.99)	<0.001	1.33 (0.96,1.85)		0.085	1.79 (1.21,2.65)		0.003
Strongly disagree	1.50 (1.25,1.79)	<0.001	1.57 (1.02,2.44)		0.042	2.24 (1.45,3.44)		<0.001
Supervisor support								
Helpful	1.00 (reference)		1.00 (reference)			1.00 (reference)		
Not helpful	0.95 (0.85,1.07)	0.380	0.89 (0.77,1.03)		0.123	1.20 (1.00,1.43)		0.044
Coworker support								
Can be relied on	1.00 (reference)		1.00 (reference)			1.00 (reference)		
Cannot be relied on	1.05 (0.97,1.14)	0.245	1.17 (1.05,1.31)		0.004	1.22 (1.05,1.43)		0.011
Decision empowerment								
Neutral to strongly agree	1.00 (reference)		1.00 (reference)			1.00 (reference)		
Disagree	1.05 (0.95,1.17)	0.346	1.28 (1.12,1.47)		<0.001	1.56 (1.41,1.73)		<0.001
Strongly disagree	1.01 (0.91,1.12)	0.827	1.27 (1.03,1.56)		0.027	1.67 (1.41,1.97)		<0.001
Patient deaths								
Never	1.00 (reference)		1.00 (reference)			1.00 (reference)		
1-2 times	1.38 (1.15,1.65)	<0.001	0.86 (0.69,1.06)		0.163	0.99 (0.88,1.12)		0.895
3 or more times	1.90 (1.70,2.13)	<0.001	0.98 (0.82,1.17)		0.801	1.12 (0.92,1.35)		0.262
Denial of family visits								
0–4 times	1.00 (reference)		1.00 (reference)			1.00 (reference)		
5 or more times	1.20 (1.05,1.37)	0.009	0.98 (0.85,1.13)		0.785	0.96 (0.80,1.15)		0.644
Short-staffing								
2 months or less	1.00 (reference)		1.00 (reference)			1.00 (reference)		
3 months or longer	1.17 (1.05,1.31)	0.006	1.19 (1.03,1.38)		0.016	1.42 (1.22,1.64)		<0.001
Work-family conflict								
Never to sometimes	1.00 (reference)		1.00 (reference)			1.00 (reference)		
Often or most of the time	1.88 (1.61,2.19)	<0.001	3.22 (2.30,4.51)		<0.001	3.15 (2.43,4.07)		<0.001
Age								
29 and under	1.35 (1.05,1.73)	0.020	0.96 (0.62,1.48)		0.857	1.81 (1.36,2.41)		<0.001
30-39	1.19 (0.99,1.42)	0.068	0.96 (0.74,1.26)		0.788	1.54 (1.21,1.96)		<0.001
40-49	1.23 (0.98,1.53)	0.069	0.98 (0.74,1.30)		0.892	1.31 (1.06,1.60)		0.011
50-59	1.25 (1.00,1.55)	0.047	1.07 (0.84,1.36)		0.593	1.32 (1.09,1.58)		0.004
60 years or older	1.00 (reference)		1.00 (reference)			1.00 (reference)		
Occupation								
Registered nurse	1.00 (reference)		1.00 (reference)			1.00 (reference)		
LPN/LVN or nursing assistant	1.22 (1.07,1.40)	0.004	1.18 (0.94,1.46)		0.147	0.93 (0.77,1.12)		0.455
Nurse practitioner or physician assistant	0.92 (0.69,1.23)	0.566	0.85 (0.61,1.20)		0.360	1.29 (0.87,1.91)		0.200
Physician	0.68 (0.55,0.83)	<0.001	0.49 (0.33,0.73)		<0.001	0.89 (0.61,1.29)		0.529
Psychologist or social worker	0.68 (0.45,1.04)	0.074	1.09 (0.69,1.74)		0.709	1.03 (0.71,1.51)		0.866
Other hands-on patient care	0.98 (0.79,1.21)	0.842	1.06 (0.86,1.30)		0.574	0.88 (0.72,1.07)		0.187
Admin in clinical area, housekeeping, or other	1.08 (0.77,1.52)	0.641	1.87 (1.45,2.41)		<0.001	1.12 (0.74,1.71)		0.584
Supervisor status								
Non-supervisor	1.00 (reference)		1.00 (reference)			1.00 (reference)		
Supervisor	1.06 (0.82,1.36)	0.660	1.06 (0.75,1.50)		0.733	0.69 (0.51,0.94)		0.018
Gender								
Man	1.00 (reference)		1.00 (reference)			1.00 (reference)		
Woman, other, non-binary	1.01 (0.89,1.14)	0.897	0.83 (0.69,0.99)		0.033	0.89 (0.77,1.03)		0.120
Race and ethnic minority								
White	1.00 (reference)		1.00 (reference)			1.00 (reference)		
Other race/ethnic minority	1.25 (1.12,1.40)	<0.001	1.02 (0.88,1.17)		0.829	0.90 (0.76,1.06)		0.210

## Discussion

This study aimed to evaluate risk for moral injury among frontline VA healthcare workers and to examine how moral injury risk is related to worker characteristics, workplace culture, COVID-19 pandemic exposures, and facility-level measures of care quality, patient satisfaction, and staff satisfaction. We found that nearly 40% of surveyed healthcare workers met our criteria for a positive MIES screen and should be considered at risk for moral injury. In inpatient, emergency, and nursing-home settings—locations that we hypothesized to be at highest risk due to routine exposure to patient deaths and high-stakes decisions—41% of workers screened positive on the MIES compared to 29% in other work locations. These rates are comparable to those found in related studies of healthcare workers conducted since the start of the COVID-19 pandemic [13–16].

The pandemic likely exacerbated existing contributors to moral injury in the healthcare workplace and also created new ones [5–8]. Consistent with research conducted to date, we found that moral injury risk was linked to pandemic-related exposures, including short-staffing, exposure to patient deaths, and denial of family visits to critically ill patients. Yet we also found that moral injury risk was linked with multiple modifiable workplace factors that have continuing relevance in the aftermath of the pandemic. These risk factors include limited involvement of frontline staff in decision-making and policy-setting, lack of meaningful leadership engagement with frontline staff, unequal distribution of risks and burdens in the workplace, and lack of work-life balance among frontline clinicians. Workplace risk factors that had direct negative impacts on individual patients, including provider-to-family communication failures and delivery of futile care, were the most distressing to interviewed workers. These events, while perhaps more common during the pandemic, are of significant concern at other times as well [32–35].

Age appeared to be significantly and strongly associated with moral injury in initial unadjusted analyses, with younger workers at greater risk. Age had a similar relationship to posttraumatic stress and burnout. Other studies conducted during the COVID-19 pandemic have found similar relationships between age and moral injury among healthcare workers [8,9,36]. Yet we found that age was not a significant risk factor after adjusting for other covariates. This suggests that the relationship between age and moral injury risk could be explained by younger workers’ greater exposure to other workplace risk factors. For example, younger age may be associated with trainee status or less decision-making authority. It is possible that younger workers are more likely to be more junior in the workplace, which could constrain their ability to influence their environment or effect change.

Our findings bolster recent research showing the potential impact of moral injury and its significant relationship to other mental health outcomes affecting worker well-being [13,14,17,18,37–40]. In line with other research conducted during the COVID-19 pandemic, we found that workers at risk for moral injury were also at significantly higher risk for posttraumatic stress, depression, and burnout. Further, we found that the predictors of these distinct outcomes were similar, so there is reason to believe that addressing the factors that contribute to moral injury could also reduce risk for posttraumatic stress, depression, and burnout.

It is striking that predictors of moral injury *and* other mental health outcomes included multiple modifiable factors linked to organizational culture. Modifiable factors such as management concern for worker health and safety, supervisor support, and coworker support were consistently stronger predictors of all outcomes than intrinsic factors like worker demographics, occupation, and supervisory status. Short-staffing—another modifiable risk factor—was a particularly strong predictor of moral injury risk as well as risk for posttraumatic stress, depression, and burnout. The relationships that we identified persisted across higher- and lower-risk work locations. Although exposures to risk factors and outcome rates were generally higher in the work locations we hypothesized to be at high risk, the associations between risk factors and outcomes remained remarkably consistent, which suggests that these findings likely generalize to a wide range of work locations.

Our findings support the conceptualization of moral injury as a culturally and institutionally propagated injury to the individual, as opposed to a mental illness. Healthcare workers can experience moral injury when placed in situations of significant personal responsibility within a context of obstinate structural constraints, often paired with high mental and physical demands. Within this context, factors related to workplace culture may impact whether a healthcare worker becomes morally injured. Our qualitative findings suggest that the relevant factors are often highly local—for instance, the perception of support from one’s own supervisor or one’s own coworkers. Our quantitative findings show that workers’ perception of how much management is concerned about workplace health and safety is highly predictive of moral injury, above and beyond all other identified risk factors. These findings align with those of Plouffe and colleagues [41], who found that strong leadership at both the organizational and supervisory levels predicted healthcare workers’ perceptions of a supportive and ethical workplace climate, which in turn were protective against moral distress. In our analyses, facility-level metrics did not consistently or significantly predict moral injury risk, but this may be, in part, because facility-level metrics were less sensitive than our survey measures to intra-organizational disparities in risk and burden, which interviewees identified as potential drivers of moral injury risk and of differential exposures among staff.

Our findings suggest that institutional betrayal—the most prevalent distressing experience reported by surveyed workers—is not only a contributor to moral injury but is perhaps also the context in which other (potentially more damaging) morally injurious acts of perpetration take place. This hypothesis has been put forward by Weber and colleagues [18] based on a latent class analysis of moral injury patterns. It is possible that institutional betrayal first wounds, and, over time, threatens to degrade the conscience. One might even conceptualize betrayal-based moral injury as an internalization of structural (or institutional) violence that, when severe or prolonged, can damage character and mental health. In our study, perpetration-based moral injury risk was relatively rare with 10% at risk compared to 30% at risk for betrayal-based moral injury. Some studies have found that perpetration-based moral injury is most closely linked to negative mental health outcomes [17,18]. Addressing the organizational risk factors that contribute to betrayal-based moral injury may be protective against perpetration-based moral injury and may mitigate the most severe moral injury outcomes.

Healthcare organizations may already have a roadmap to addressing identified organizational risk factors. The high reliability organization (HRO) model has three pillars with clear parallels to the moral injury risk factors identified in this study [42]. The first pillar is leadership commitment, which refers to leadership’s visible investment in healthcare safety and reliability, as consistently reflected in leaders’ decisions, actions, and communication. The second is safety culture, reflected in the organization’s commitment to learn from all mistakes and act continuously to reduce harm. The third is continuous process improvement, reflected in the organization’s empowerment of frontline teams to make changes and take actions based on lessons learned. HRO also embraces “just culture” principles, which entail recognizing and learning from errors and failures rather than placing blame. In a just culture model, failures are identified and embraced in order to make changes to processes and policies that reduce the likelihood of continued failures. Each of these HRO principles aligns with and addresses multiple organizational factors that we identified as closely linked to moral injury risk. To date, there is evidence that embracing HRO principles improves patient safety outcomes, but less is known about its impact on healthcare workers or on moral injury [43].

At the time we conducted our study, VA’s national leadership had already committed to HRO principles and rolled out HRO training nationwide. Our study nonetheless identified concerning rates of moral injury and other negative mental health outcomes—likely exacerbated by the COVID-19 pandemic. The journey to becoming a high reliability organization can be a long and difficult one and is perhaps always a work in progress. Our findings nonetheless point toward HRO values and practices as an important strategy to mitigate some of the most significant organizational risk factors for moral injury.

Consistent with HRO principles, our study suggests the importance of involving staff at all levels of the organization in discussing high-stakes decisions that will affect them and their workplace—the “nothing about me without me” approach. Other practices that may mitigate moral injury risk include creating consistent, open, clear channels of communication that allow messages from the frontline to reach the top of the organization *and* vice-versa, practicing leadership rounding to improve leaders’ understanding of the daily work of frontline teams, and collaborating to understand how existing processes and policies may contribute to safety risks and moral conflict. Healthcare leaders can create supportive environments for teams to proactively engage with the complex moral and ethical matters that can arise in the practice of healthcare. At routine staff meetings and as needed, managers can consider building in time for facilitated discussions about emotional and moral challenges that may arise in the workplace and how to address them together. Our research team has published additional guidance for healthcare organizations available through Healthforce Center at UCSF [healthforce.ucsf.edu; 44].

## Limitations

This study was conducted exclusively with workers employed by the VA. Although VA is a large and diverse healthcare system spanning the United States, it is different from private and non-profit healthcare systems in significant ways. As a public healthcare system, VA is largely exempt from selected drivers of moral injury in the private sector, including prohibitive costs of care, the private insurance system, and differential access based on socioeconomic status. Additional research is necessary to elucidate how these and related factors may contribute to moral injury in private-sector healthcare settings.

The response rate for our survey was 27%, and it is possible that workers who were more impacted by the pandemic were more likely to respond to our e-mail invitation. We deliberately focused on workers in higher-risk settings so the overall prevalence of moral injury in our sample is likely to be higher than the prevalence in the general population of healthcare workers.

There are also limitations in our survey measures. We used the MIES, which, as mentioned, does not measure moral injury symptoms but rather exposure to potentially morally injurious events and distress related to those exposures. MIES is a proxy for measuring moral injury risk and was the best tool available at the time of our study. Future studies should complement the MIES with newer, more symptom-sensitive measures of moral injury such the Moral Injury and Distress Scale [45,46]. Another limitation in our study is the use of individual-level surveys to measure workplace culture variables. Future research should incorporate a multi-level analysis that assesses moral injury risk and covariates of interest at the unit level.

Finally, we attempted to assess the relationship between moral injury risk at the facility-level and facility-wide metrics related to care quality and safety. Our analysis did not show any consistent patterns, but this may be because there was limited variation in facility-level metrics among the 21 facilities that participated. A larger sample of facilities may be needed to detect drivers of facility-level differences in moral injury risk. Ideally, future research would include an adequately powered, facility-level analysis of moral injury risk.

## Conclusion

Healthcare workers are at significant risk for moral injury, as well as posttraumatic stress, depression, and burnout. Risk for moral injury is strongly associated with multiple modifiable risk factors linked to organizational health and culture, including management’s support for workplace health and safety, supervisory support, and coworker support. By embracing HRO principles and addressing underlying contributors to moral distress in the workplace, healthcare organizations can reduce their workers’ risk of moral injury.
